# Evaluating Trans‐Fatty Acids Labelling in Packaged Foods Sold in Brazil Before and After National Policy Changes

**DOI:** 10.1111/jhn.70253

**Published:** 2026-05-10

**Authors:** Beatriz Vasconcellos de Barros, Mariana Vieira dos Santos Kraemer, Lana Vanderlee, Elisa Milano, Tailane Scapin, Greyce Luci Bernardo, Maria Cecília Cury Chaddad, Paula Lazzarin Uggioni, Rossana Pacheco da Costa Proença, Ana Carolina Fernandes

**Affiliations:** ^1^ Nutrition in Foodservice Research Center, Health Sciences Center Federal University of Santa Catarina Florianópolis Brazil; ^2^ Postgraduate Program in Nutrition, Health Sciences Center Federal University of Santa Catarina Florianópolis Brazil; ^3^ École de nutrition, Centre Nutrition, santé et société (NUTRISS), Institut sur la nutrition et les aliments fonctionnels (INAF) Université Laval Québec Québec Canada; ^4^ Institute for Health Transformation, Global Centre for Preventive Health and Nutrition (GLOBE), School of Health and Social Development, Faculty of Health Deakin University Geelong Victoria Australia; ^5^ Movimento Põe no Rótulo São Paulo Brazil

**Keywords:** census method, food labelling, industrialised foods, partially hydrogenated oil

## Abstract

**Introduction:**

The elimination of industrial trans‐fatty acids (i‐TFA) is a global public health priority. In Brazil, food regulatory changes were implemented in 2012, limiting the use of TFA‐free claims and in 2019, restricting i‐TFA content in food. This study analysed i‐TFA declarations on labels of packaged foods sold in Brazil in 2010, 2013 and 2020, before, during and after food policy changes.

**Methods:**

This repeated cross‐sectional study analysed labels of packaged foods from an outlet of a large supermarket chain in Brazil (*n* = 2327 products in 2010; *n* = 3176 in 2013, *n* = 4397 in 2020). The i‐TFA terms listed in the ingredient list, content of trans‐fatty acids (TFA) declared on nutrition information panels (NIPs), and TFA‐free claims were examined. Descriptive and comparative analysis over time were conducted using binary and multinomial logistic regressions.

**Results:**

The percentage of foods containing potential i‐TFA ingredients was 50.6% in 2010, 36.4% in 2013 and 28.5% in 2020. Overall, the likelihood that NIPs declared TFA decreased over time (OR: 0.46; 95%CI: 0.40; 0.53, *p* < 0.001), but this was not consistent across all food groups and years. The likelihood of using TFA‐free claims also decreased (OR: 0.12; 95%CI: 0.10; 0.15, *p* < 0.001) over time. However, in 2020, 24.1% of foods labelled as containing 0 g TFA in their NIPs still listed ingredients that could be sources of i‐TFA.

**Conclusion:**

This is the largest study to analyse TFA labelling in Brazil, informing regulatory discussions and offering a basis for assessing compliance with TFA‐focused labelling regulations. Our findings suggest that the 2019 regulation on i‐TFA restrictions contributed to amplifying and sustaining the impact of the 2012 labelling regulations in reducing i‐TFA packaged food sold at the retail level.

## Summary

- The implementation of labelling (2012) and content (2019) regulations contributed to a marked decline in the presence of industrial trans‐fatty acids (i‐TFA) in packaged foods sold in Brazil.
- In 2020, approximately one‐quarter of products declaring ‘0 g TFA’ still listed potential i‐TFA ingredients, underscoring the importance of continuous monitoring and enforcement.
- Practitioners should be aware of inconsistencies between ingredient lists and nutrition information panels, and guide consumers accordingly when interpreting TFA‐related labelling.

## 1 Introduction

Oils and fats have important functions in packaged foods [1]. Often, the use of oils and fats requires industrial modification processes to alter their composition and provide structure to the packaged foods [2]. One common method of modification is the partial hydrogenation, which creates partially hydrogenated oils (PHO), primarily generating elaidic acid [3] and conventionally known as industrial trans‐fatty acids (i‐TFA). Besides this industrial form, trans‐fatty acids (TFA) can be produced through biohydrogenation in the intestines of ruminant animals [4], resulting in natural TFA, such as rumenic acid.

Fats with i‐TFA have been used extensively in industrialised packaged foods since 1920 [5]. However, since the early 1990, studies started to associate i‐TFA consumption with negative health impacts, in particular relating to cardiovascular diseases [6, 7, 8]. Considering the well‐established harmful impacts of the i‐TFA on human health, in 2018, the World Health Organization (WHO) launched the REPLACE program outlining strategies aiming for countries to adopt policies to limit i‐TFA up to 1% of the daily energy intake or eliminate i‐TFA [9]. In Brazil, a mandatory regulation from 2012 imposed more restrictive rules for using TFA‐free claims on packaged foods. In 2019, another mandatory regulation was passed limiting i‐TFA to 2 g per 100 g of total fat in all commercialised foods and refined oils, with mandatory implementation by July 2021. The regulation also banned the production, import, use and sale of PHO, the main source of i‐TFA, by December 2023 [10].

A systematic review of TFA intake across 29 countries with national surveys or population‐based data found intake levels ranging from 0.3% to 4.2% of daily energy. Among 20 countries with longitudinal or cross‐sectional data (1987–2017), only seven reported intakes below 1% of daily energy. In Brazil, Canada, Costa Rica and the United States, surveys up until 2010 showed TFA intake remained above 1% [11]. The consumption above this threshold is associated with increased risk of coronary heart disease and mortality [9], among other diseases, increasing the public health burden.

One of the most effective ways to identify if a packaged food is a source of TFA (industrially produced or natural) is through the food labels, which are the primary communication channel between consumers and the food manufacturer [12]. Studies have observed a decrease in the use of terms that identify potential sources of i‐TFA in the ingredient lists and TFA levels on nutrition information panels (NIPs) over time, but trends have typically varied by food group, with some categories showing increases [13, 14, 15, 16]. At the time of data collection, none of the studied countries had i‐TFA restrictions in force, which were later passed in 2018 in Canada, 2019 in Brazil and in 2021 in Estonia and the Netherlands.

Continuous monitoring of the presence of i‐TFA in the food supply permits the evaluation of the effects of food policy changes. The current study aimed to compare the prevalence of i‐TFA and TFA declared on food labels of packaged foods sold in Brazil in 2010, 2013 and 2020, in the context of food policy changes.

## 2 Materials and Methods

We performed a comparative analysis using a repeated cross‐sectional design with food label censuses carried out in 2010, 2013 and 2020 in two stores from a large supermarket chain in Brazil.

### 2.1 Data Collection

Food censuses conducted in 2010, 2013 and 2020 collected data from all packaged foods available for sale from one large supermarket chain in Brazil. Data collection was conducted in two different supermarkets from the same chain in southern Brazil in 2010 and 2013, and at the same store location in 2013 and 2020. The stores were intentionally chosen from one of the 15 largest chains by market share in Brazil in all years of the study, according to the ranking of the Brazilian Supermarket Association [17, 18]. At least 69% of the foods collected in 2010, 70% in 2013 and 92% in 2020 were supplied nationwide. Permission for data collection was obtained from store managers.

All packaged foods subject to Brazilian nutrition labelling regulation [19] and with the potential to contain i‐TFA in their composition were included in this study, totalling 2327 foods collected in 2010, 3176 in 2013 and 4397 in 2020. Foods covered by different regulations (i.e., food for babies) or those that did not require mandatory nutrition labelling (i.e., sliced cheese, packed and labelled in‐store) were not included. Variations of the same type of food, such as different packaging sizes, were considered as different products. Packaged foods that do not contain ingredients that are potential sources of oils and fats (e.g., canned fruits) and single‐ingredient foods that have no potential to contain TFA were excluded from the analysis. Foods from all three data collections were classified into eight food groups based on the Brazilian regulation [20]. If a type of food within each food group (e.g., biscuits and pizza dough) did not contain at least one product with added fat in its composition, this type of food was excluded. This analysis was done for each food census individually, implying that the types of foods included in the analysis may have changed between the years. This methodology was consistent across all 3 years of the food label census.

Data collection were conducted by trained researchers. The instrument used to collect data was updated over the years to improve data collection. In May 2010, data were collected using a paper form. Data entry was done twice into separate databases using Microsoft Excel, followed by error checking for typos or inconsistencies in the ingredient lists and NIPs. From October to December 2013, data collection were conducted electronically using EpiCollect Plus software on Samsung Galaxy Note 8.0 tablets, with all sides of the packaging photographed. Agreement between entered data and photographs was assessed on a random selection of 10% of products using a weighted kappa test, which indicated 0.99 reliability. In November 2020, a smartphone application developed by The George Institute's FoodSwitch program, Australia, was used to scan barcodes and photograph all label information. A group of trained researchers transcribed the information contained in the photographs, using the FoodSwitch system. Data entry protocols are described in Dunford et al. [21] and Dunford et al. [22]. Agreement between entered data and photographs was also assessed on a random selection of 10% of products using a weighted kappa test, which indicated 0.95 reliability.

### 2.2 Data Analysis

The same food groups were included in the analysis in 2010, 2013 and 2020, which are described in Table 1. The data analyses involved: (1) identifying potential i‐TFA ingredients by screening all fat‐related terms in the ingredient lists, (2) examining total TFA content listed in the NIPs and (3) checking for TFA‐free claims on the labels (which considers the total TFA content). At least two researchers independently analysed the ingredient lists (three in 2013, and two in 2010 and 2020), with a third researcher resolving any classification disagreements.

**Table 1:** Frequencies of packaged foods that were potential sources of trans‐fatty acids in 2010, 2013 and 2020, stratified by food groups.

Group	Description	Examples of food items	2010	2013	2020
			*N* (%)	*N* (%)	*N* (%)
A	Bakery goods, bread, cereals and related foods	Salty crackers and cakes without filling	724 (31)	801 (25)	897 (20)
B	Milk and dairy	Dairy drinks and ice cream powder mix	375 (16)	327 (10)	727 (17)
C	Meats, eggs and seafood	Sausages, meat pastes, burgers and chicken nuggets	97 (4)	461 (15)	652 (15)
D	Oils, fats and nuts	Mayonnaise and salad dressings	77 (3)	141 (4)	270 (6)
E	Sugars, sugary foods and snacks	Sweet biscuits, cakes with filling and ice cream	753 (33)	1146 (36)	1460 (33)
F	Instant meals and recipe boosters	Ready‐to‐cook and ready‐to‐eat dishes and sauce mix	301 (13)	300 (10)	391 (9)
Total			2327 (100)	3176 (100)	4397 (100)

Ingredients indicating hydrogenation (e.g., presence of the words ‘partially hydrogenated’) were classified as specific i‐TFA terms, while ingredients potentially containing i‐TFA (e.g., margarine, vegetable fat) were classified as ‘alternative terms’. If both specific and alternative terms appeared, the food was classified based on the specific term for certainty. Throughout the paper, the term TFA was only used when referring to total TFA (e.g., when referring to TFA‐free claim or TFA content on NIP, that consider total TFA content); to natural TFA sources or when it was not possible to distinguish between TFA and i‐TFA. Foods with compound ingredients without opening their composition were classified as containing alternative terms for i‐TFA if similar foods in the dataset identified the same ingredient as a source of i‐TFA. Packaged foods were considered potential sources of i‐TFA when at least one alternative term was found in the ingredient list. Foods were classified as ‘false negatives’ if they contained specific or alternative terms in the ingredient lists but made a TFA‐free claim or listed 0 g TFA in the NIPs. Data are presented descriptively as absolute and relative frequencies, overall and stratified by food groups, with comparative analyses between 2010, 2013 and 2020s. Binary and multinomial logistic regression models were used to compare TFA labelling and claims across these years, with results reported as odds ratios (OR) at a 95% confidence level. All analyses were done using R version 4.3.3.

## 3 Results

### 3.1 Analysis of the Presence of Potential i‐TFA

Table 1 shows the number of foods that were potential sources of i‐TFA analysed per food group in 2010, 2013 and 2020. There was an increase in the number of products included in the study: a 36.4% relative increase between 2010 and 2013, and a 38.4% relative increase between 2013 and 2020, representing an overall increase of 88.9% between 2010 and 2020.

Between 2010 and 2013, the largest increase in food items was in meats, eggs and seafood (Group C), driven by a greater variety of burgers, chicken nuggets and sausages. By 2020, the greatest growth was in milk and dairy (Group B), mainly due to more vegetable beverages and yogurt flavours. Oils, fats and nuts (Group D) also had new additions, particularly margarine, peanut butter and bacon.

**Figure 1:**
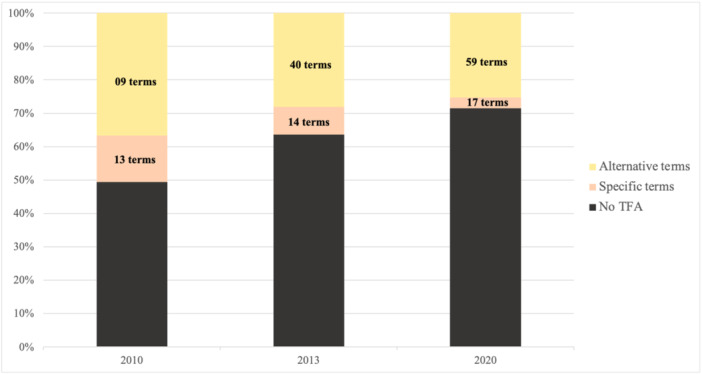
Frequencies of alternative and specific terms of industrial trans‐fatty acids (i‐TFA) in 2010, 2013 and 2020.

Across all years, 108 terms for i‐TFA (specific and alternative) were identified (Supporting Information: Tables S1 and S2). Figure 1 illustrates this data.

Despite an increased number of specific terms, their percentage relative to the total number of nomenclatures decreased (27.5% in 2010, 22.9% in 2013 and 11.1% in 2020). Consequently, both the number and percentage of alternative terms increased over the years (72.5% in 2010, 77.1% in 2013 and 88.9% in 2020).

As shown in Table 2, 50.6% (*n* = 1177) of the packaged foods analysed in 2010 declared alternative terms of i‐TFA on the ingredient list. This percentage decreased to 36.4% (*n* = 1157) in 2013 and to 28.5% (*n* = 1275) in 2020. Overall, there was a 22.1% percentage point (pp) decrease in the proportion of foods that were potential sources of i‐TFA between 2010 and 2020.

**Table 2:** Prevalence of false negatives and packaged foods declaring TFA (total, natural and industrial) on ingredients list, nutrition information panels and nutrition claims in 2010, 2013 and 2020.

Years	*n*	Foods declaring i‐TFA on Ingredients list (*N* = 9900)			Foods containing TFA declaration on nutrition Information panels (*N* = 9464)						Foods containing TFA‐free Nutrition claims (*N* = 9900)			
					> 0 g TFA (total) (%)	> 0 g i‐TFA (industrial) (%)	> 0 g natural TFA (%)	False negatives			TFA free claims	False negatives		
		Total (%)	Specific terms (%)	Alternative terms (%)				Total (%)	Specific terms (%)	Alternative terms (%)		Total (%)	Specific terms	Alternative terms
*All food groups*														
2010	2327	1177 (50.6)	324 (13.9)	853 (37.0)	421 (18.1)	307 (13.2)	114 (4.9)	863 (37.1)	164 (19.0)	699 (81.0)	517 (16.3)	300 (12.9)	51 (17.0)	249 (83.0)
2013	3176	1157 (36.4)	265 (8.3)	892 (28.1)	376 (11.8)	270 (8.5)	106 (3.3)	845 (26.6)	142 (16.8)	703 (83.2)	435 (18.7)	176 (5.5)	35 (19.9)	141 (80.1)
2020	4397	1252 (28.5)	139 (3.2)	1113 (25.3)	399 (9.1)	202 (4.6)	197 (4.5)	1059 (24.1)	92 (8.7)	967 (91.3)	151 (3.4)	19 (0.4)	6 (31.6)	13 (68.4)
*Group A—Bakery goods, bread, cereals and related products*														
2010	724	426 (58.8)	105 (14.5)	321 (44.3)	138 (19.1)	123 (17.0)	15 (2.1)	299 (41.3)	62 (20.7)	237 (79.3)	240 (30.0)	128 (17.7)	25 (19.5)	103 (80.5)
2013	801	283 (35.3)	79 (10.0)	204 (25.5)	87 (10.9)	83 (10.4)	4 (0.5)	196 (24.5)	31 (15.8)	165 (84.2)	180 (20.0)	54 (6.7)	6 (11.1)	48 (88.9)
2020	897	273 (30.4)	35 (3.9)	238 (26.5)	62 (6.9)	60 (6.7)	2 (0.2)	220 (24.5)	14 (6.4)	206 (93.6)	79 (10.9)	8 (0.9)	2 (25.0)	6 (75.0)
*Group B—Milk and dairy products*														
2010	375	23 (6.1)	6 (1.6)	17 (4.5)	55 (14.7)	5 (1.3)	50 (13.3)	18 (4.8)	4 (22.2	14 (77.8)	0 (0.0)	0 (0.0)	0 (0.0)	0 (0.0)
2013	327	19 (5.8)	6 (1.8)	13 (4.0)	31 (9.5)	0 (0.0)	31 (9.5)	19 (5.8)	6 (31.6)	13 (68.4)	5 (0.7)	0 (0.0)	0 (0.0)	0 (0.0)
2020	727	29 (4.0)	0 (0.0)	29 (4.0)	92 (12.5)	2 (0.3)	90 (12.4)	27 (3.7)	0 (0.0)	27 (100.0)	0 (0.0)	0 (0.0)	0 (0.0)	0 (0.0)
*Group C—Meats, eggs and seafood products*														
2010	97	43 (44.3)	3 (3.1)	40 (41.2)	24 (24.7)	11 (11.3)	13 (13.4)	32 (33.0)	0 (0.0)	32 (100.0)	22 (4.8)	11 (11.3)	0 (0.0)	11 (100.0)
2013	461	32 (6.9)	5 (1.1)	27 (6.0)	22 (5.0)	1 (0.2)	21 (4.6)	30 (6.5)	4 (13.3)	26 (86.7)	10 (1.5)	3 (0.6)	3 (100.0)	0 (0.0)
2020	652	42 (6.4)	1 (0.1)	41 (6.3)	62 (9.5)	6 (0.9)	56 (8.6)	37 (5.7)	1 (2.7)	36 (97.3)	8 (8.2)	0 (0.0)	0 (0.0)	0 (0.0)
*Group D—oils, fats and nuts*														
2010	77	31 (40.3)	17 (22.1)	14 (18.2)	13 (16.9)	4 (5.2)	9 (11.7)	25 (32.5)	13 (52.0)	12 (48.0)	24 (17.0)	0 (0.0)	0 (0.0)	0 (0.0)
2013	141	24 (17.0)	14 (9.9)	10 (7.1)	1 (0.7)	1 (0.7)	0 (0.0)	23 (16.3)	13 (56.5)	10 (43.5)	41 (16.2)	3 (2.1)	2 (66.7)	1 (33.3)
2020	270	47 (17.4)	7 (2.3)	40 (14.8)	22 (8.1)	5 (1.9)	17 (6.3)	44 (16.3)	5 (11.4)	39 (88.6)	6 (7.8)	0 (0.0)	0 (0.0)	0 (0.0)
*Group E—Sugars, sugary foods and snacks*														
2010	753	504 (66.9)	168 (22.3)	336 (44.6)	146 (19.4)	138 (18.3)	8 (1.1)	365 (48.5)	65 (17.8)	300 (82.2)	199 (17.4)	141 (18.7)	26 (18.4)	115 (81.6)
2013	1146	633 (55.2)	125 (10.9)	508 (44.3)	163 (14.2)	125 (10.9)	38 (3.3)	481 (42.0)	70 (14.5)	411 (85.5)	176 (12.1)	100 (8.7)	17 (17.0)	83 (83.0)
2020	1460	740 (50.7)	83 (5.7)	657 (45.0)	128 (8.8)	103 (7.1)	25 (1.7)	632 (43.3)	59 (9.3)	573 (90.7)	44 (5.8)	9 (0.6)	4 (44.4)	5 (55.6)
*Group F—Instant meals and recipe boosters*														
2010	301	150 (49.8)	25 (8.3)	125 (41.5)	45 (15.0)	26 (8.6)	19 (6.3)	124 (41.2)	20 (16.1)	104 (83.9)	32 (10.7)	20 (6.6)	0 (0.0)	20 (100.0)
2013	300	166 (55.3)	36 (12.0)	130 (43.3)	72 (24.0)	60 (20.0)	12 (4.0)	96 (32.0)	18 (18.8)	78 (81.3)	23 (5.9)	16 (5.3)	7 (43.7)	9 (56.3)
2020	391	121 (31.0)	13 (3.3)	108 (27.6)	33 (8.4)	26 (6.6)	7 (1.8)	99 (25.3)	13 (13.1)	86 (86.9)	14 (4.7)	2 (0.5)	0 (0.0)	2 (100.0)

### 3.2 Comparative Analysis

The data in Table 3 reflect changes in the likelihood of packaged foods declaring i‐TFA terms on ingredient lists. Overall, there was a decrease of 44% between 2010 and 2013, 31% between 2013 and 2020 and 61% between 2010 and 2020 (all *p* < 0.001). Decreases were inconsistent across food groups and years. For instance, for milk and dairy (Group B), there was no change in the likelihood of declaring i‐TFA over time, while instant meals and recipe boosters (Group F) only improved after 2013. Meats, eggs and seafood (Group C) and oils, fats and nuts (Group D) showed no significant changes after 2013.

**Table 3:** Logistic regressions of packaged foods with trans‐fatty acids (TFA) between 2010, 2013 and 2020 in Brazil, as determined by industrial trans‐fatty acids (i‐TFA) declaration on the ingredients list, TFA declaration on nutrition information panels and trans‐fat‐free nutrition claims.

Food groups years	Foods declaring i‐TFA on the ingredients list (*N* = 9900)					Foods containing > 0 g TFA declaration on the nutrition information panels (*N* = 9464)				Foods containing TFA‐free nutrition claims (*N* = 9900)	
	All labels	*X* ^2^ *p*‐value	Use of specific terms**	Use of alternative terms**	*X* ^2^ *p*‐value	TFA > 0 g	*X* ^2^ *p*‐value	i‐TFA > 0 g (*n* = 779)	*X* ^2^ *p*‐value	All labels	*X* ^2^ *p*‐value
	OR 95% CI		OR 95%CI	OR 95% CI		OR 95% CI		OR 95% CI		OR 95% CI	
*All food groups*											
		**< 0.001**			**< 0.001**		**< 0.001**		**< 0.001**		**< 0.001**
2013 versus 2010^a^	0.56 (0.50; 0.62)		0.47 (0.39; 0.56)	0.59 (0.53; 0.67)		0.69 (0.59; 0.80)		0.69 (0.58; 0.82)		0.56 (0.48; 0.64)	
2020 versus 2010^a^	0.39 (0.35; 0.43)		0.16 (0.13; 0.19)	0.48 (0.43; 0.53)		0.46 (0.40; 0.53)		0.32 (0.27; 0.39)		0.12 (0.10; 0.15)	
2020 versus 2013^b^	0.69 (0.63; 0.77)		0.34 (0.27; 0.42)	0.80 (0.72; 0.89)		0.67 (0.57; 0.77)		0.47 (0.39; 0.56)		0.22 (0.18; 0.27)	
*Group A—Bakery goods, bread, cereals and related products*											
		**< 0.001**			**< 0.001**		**< 0.001**		**< 0.001**		**< 0.001**
2013 versus 2010^a^	0.38 (0.31; 0.47)		0.43 (0.31; 0.60)	0.37 (0.29; 0.46)		0.52 (0.39; 0.69)		0.56 (0.42; 0.76)		0.58 (0.46; 0.73)	
2020 versus 2010^a^	0.31 (0.25; 0.37)		0.16 (0.11; 0.24)	0.35 (0.28; 0.44)		0.31 (0.23; 0.43)		0.35 (0.25; 0.48)		0.19 (0.15; 0.26)	
2020 versus 2013^b^	0.80 (0.65; 0.98)		0.37 (0.24; 0.56)	0.97 (0.77; 1.21)		0.61 (0.43; 0.85)		0.62 (0.43; 0.87)		0.33 (0.25; 0.44)	
*Group B—Milk and dairy products*											
		0.217			**0.001**		0.096		**0.020**		**< 0.001**
2013 versus 2010^a^	0.94 (0.50; 1.76)		*	*		0.60 (0.37; 0.96)		*		*	
2020 versus 2010^a^	0.64 (0.36; 1.12)		*	*		0.85 (0.59; 1.22)		*		*	
2020 versus 2013^b^	0.67 (0.37; 1.24)		*	*		1.40 (0.92; 2.18)		*		*	
*Group C—Meats, eggs and seafood products*											
		**< 0.001**			**< 0.001**		**0.001**		**< 0.001**		**< 0.001**
2013 versus 2010^a^	0.09 (0.05; 0.16)		0.21 (0.05; 1.90)	0.08 (0.05; 0.15)		0.44 (0.23; 0.83)		0.04 (0.00; 0.24)		0.08 (0.03; 0.16)	
2020 versus 2010^a^	0.09 (0.05; 0.14)		0.03 (0.00; 0.29)	0.09 (0.05; 0.15)		0.35 (0.21; 0.60)		0.08 (0.03; 0.21)		0.04 (0.02; 0.09)	
2020 versus 2013^b^	0.92 (0.57; 1.49)		0.14 (0.02; 1.21)	1.07 (0.65; 1.76)		0.79 (0.48; 1.36)		1.74 (0.29; 33.03)		0.56 (0.21; 1.43)	
*Group D—Oils, fats and nuts*											
		**< 0.001**			**< 0.001**		**< 0.001**		0.115		**< 0.001**
2013 versus 2010^a^	0.30 (0.16; 0.57)		0.32 (0.15; 0.71)	0.28 (0.12; 0.68)		0.04 (0.00; 0.19)		0.13 (0.01; 0.92)		0.90 (0.50; 1.67)	
2020 versus 2010^a^	0.31 (0.18; 0.54)		0.08 (0.03; 0.22)	0.59 (0.30; 1.17)		0.44 (0.21; 0.94)		0.35 (0.09; 1.43)		0.05 (0.02; 0.12)	
2020 versus 2013^b^	1.03 (0.60; 1.79)		0.26 (0.10; 0.67)	2.10 (1.01; 4.35)		12.25 (2.53; 220.61)		2.60 (0.41; 50.16)		0.05 (0.02; 0.12)	
*Group E—sugars, sugary foods and snacks*											
		**< 0.001**			**< 0.001**		**< 0.001**		**< 0.001**		**< 0.001**
2013 versus 2010^a^	0.61 (0.50; 0.74)		0.36 (0.27; 0.48)	0.73 (0.60; 0.90)		0.71 (0.56; 0.91)		0.56 (0.43; 0.73)		0.50 (0.40; 0.63)	
2020 versus 2010^a^	0.51 (0.42; 0.61)		0.17 (0.13; 0.23)	0.68 (0.56; 0.82)		0.40 (0.31; 0.52)		0.34 (0.26; 0.45)		0.09 (0.06; 0.12)	
2020 versus 2013^b^	0.83 (0.71; 0.97)		0.47 (0.35; 0.64)	0.92 (0.78; 1.08)		0.56 (0.44; 0.72)		0.60 (0.46; 0.79)		0.17 (0.12; 0.24)	
*Group F—instant meals and recipe boosters*											
		**< 0.001**			**< 0.001**		**< 0.001**		**< 0.001**		**< 0.001**
2013 versus 2010^a^	1.25 (0.90; 1.72)		1.62 (0.93; 2.84)	1.17 (0.83; 1.64)		1.90 (1.26; 2.90)		2.80 (1.73; 4.64)		0.70 (0.39; 1.22)	
2020 versus 2010^a^	0.45 (0.33; 0.61)		0.29 (0.14; 0.58)	0.48 (0.35; 0.67)		0.55 (0.34; 0.88)		0.79 (0.45; 1.39)		0.31 (0.16; 0.58)	
2020 versus 2013^b^	0.36 (0.26; 0.49)		0.18 (0.09; 0.35)	0.41 (0.30; 0.57)		0.29 (0.18; 0.45)		0.28 (0.17; 0.45)		0.45 (0.22; 0.87)	

*Note:* Bold *p*‐values indicate statistical significance (*p* < 0.05).

^a^ 2010 was used as a reference.

^b^ 2013 was used as a reference.

* Insufficient number of items in the category for analysis of statistical significance.

** Results from multinomial logistic regression (reference group = no terms).

There was a general decrease in the declaration of both specific and alternative i‐TFA terms in ingredient lists over time across all packaged foods (Table 3). However, when comparing the likelihood of using specific or alternative terms more often by food groups, there were no meaningful changes in the use of alternative terms in most groups. A significant decline in specific i‐TFA terms declaration was noted in meats, eggs and seafood (Group C) from 2020 to 2010, while instant meals and recipe boosters (Group F) only showed a reduction after 2013. Meanwhile, the likelihood of declaring alternative terms did not change between 2013 and 2020 for bakery goods, bread, cereals and related (Groups A), meats, eggs and seafood (Groups C) and oils, fats and nuts (Group D). For this last group, this likelihood also remained unchanged for the overall comparison between 2020 and 2010.

Considering all food groups (Table 3), the likelihood of declaring i‐TFA in the NIPs decreased by 31% between 2010 and 2013, 68% between 2013 and 2020, and 53% overall (*p* < 0.001). However, when analysing the food groups individually, only bakery goods, bread, cereals and related (Groups A), and sugars, sugary foods and snacks (Group E) followed this decrease consistently over time. There was no difference in oils, fats and nuts (Group D). Also, there was no difference for meats, eggs and seafood products (Group C) between 2020 and 2013, or for instant meals and recipe boosters (Group F) between 2020 and 2010.

There was a lower likelihood of including TFA‐free nutrition claims throughout all the years of analysis, decreasing by 44% between 2010 and 2013, 88% between 2013 and 2020 and 78% overall (*p* < 0.001), except for meats, eggs and seafood products (Groups C) (between 2013 and 2020), oils, fats and nuts (Group D) (between 2010 and 2013) and for instant meals and recipe boosters (Group F) (between 2010 and 2013).

### 3.3 False Negatives Analysis

Trans‐fat reduction trends varied significantly across food categories. While most groups showed consistent declines in false‐negative TFA declarations, instant meals and recipe boosters (Group F) exhibited a distinct pattern (Supporting Information: Table S3). Unlike other categories, where reductions began by 2013, Group F showed no significant decrease in false negatives until after 2013 (OR 0.36 between 2020 and 2013). This group also stood out for its initially higher prevalence of i‐TFA declarations (OR 1.90–2.80 between 2010 and 2013) and the smallest use of TFA‐free claims in 2010. In contrast, bakery products (Group A), oils/fats (Group D) and snacks (Group E) demonstrated early progress in reducing false negatives (between 2010 and 2013), though these improvements plateaued thereafter. The use of alternative i‐TFA terms increased markedly after 2013 across most categories (*p* < 0.001). The prevalence of TFA‐free claims among products containing i‐TFA ingredients decreased 93% overall between 2010 and 2020, with Group F again being the exception during the initial study period.

## 4 Discussion

### 4.1 Ingredient Lists

Our study demonstrates a significant decline in both specific and alternative i‐TFA term declarations in Brazilian packaged foods between 2010 and 2020. However, this reduction was not uniform across all food categories. While bakery products (Group A) showed marked improvement, dairy (Group B), meat/egg/seafood (Group C) and oil/fat/nut (Group D) categories exhibited persistent i‐TFA labelling practices throughout the study period.

The results are similar to a previous study monitoring the prevalence of TFA declaration in Brazilian packaged foods comparing 2010 and 2013 [16], which also concluded that there were reductions in the number of foods with i‐TFA declaration in the ingredient list, but this was not consistent across all food groups. It was noted that, despite the decrease in foods that declared specific and alternative i‐TFA terms, this reduction was only significant for bakery goods, bread, cereals and related products (Group A), and there was an increase in the proportion for instant meals and recipe boosters (Group F) [16]. The current study adds to this discussion by including comparisons with 2020 in the analysis, by analysing the likelihood of foods to have TFA information along with the prevalence and advances the regulatory discussion after the i‐TFA prohibition.

These findings corroborate earlier Brazilian research [16] while expanding the temporal scope to include 2020 data. The accelerated decline observed strongly suggests the positive impact of Brazil's 2012 and 2019 mandatory regulations. Similar patterns have been documented globally: in Slovenia [13], ‘partially hydrogenated fat’ declarations dropped from 6% to 2% overall (though increasing in some bakery items), while Canadian data [14] showed parallel reductions in both ‘partially hydrogenated fat’ (0.8% to 0.2%) and generic ‘hydrogenated fat’ terms (5.0% to 2.4%), but this reduction did not occur in all food groups.

By 2022, the WHO reported 60 countries (43% global population) had implemented i‐TFA policies, either through 2 g/100 g fat limits or PHO bans [23]. Brazil's 2019 prohibition policy [10] appears effective, with steeper declines in PHO use during 2013–2020 versus 2010–2013. However, the full impact may exceed our 2020 findings as manufacturers had until 2021 for compliance, with complete elimination expected by 2023. These results support evidence that prohibition policies most effectively reduce PHO use [24].

The use of alternative terms makes it harder for consumers and researchers to identify i‐TFA sources in food labels, like ‘vegetable fat’—the most present in 2020 (25.3%). Gas chromatography analyses confirm these terms often mask actual i‐TFA content, as demonstrated in Brazilian cookies and bread listing ‘vegetable fat’ but containing i‐TFA [25]. While alternative term usage declined overall, this trend varied across food groups, maintaining consistent i‐TFA exposure risks for consumers [16, 26].

Critical regulatory gaps persist in labelling requirements. In Brazil, as in several other countries around the world, there is no specific regulation standardising the terminology used to indicate processed oils and fats in ingredient lists for all types of packaged foods. While Brazil's 2021 regulation improved disclosure for standalone oils/fats (requiring process specification like hydrogenation or interesterification [27]), these requirements don't extend to composite foods. Analysis of 2020 data reveals most specific terms (77%) lacked processing/sourcing details, leaving consumers unable to distinguish between i‐TFA‐containing and alternatives [28]. Most ingredient terms lacked processing/sourcing details: only 6.5% specified partial hydrogenation, 16.5% listed tropical oils, while 77% remained unspecified. As such, there is a chance that a share of the packaged foods that used specific terms in 2020 was derived from interesterification or fully hydrogenated processing, which do not contain i‐TFA [28]. However, given that precise information on processing was not available to consumers, it is relevant to consider that these packaged foods might still be a source of i‐TFA.

### 4.2 NIPs

NIPs revealed declining TFA declarations (> 0 g) across all study years, suggesting reduced TFA content in Brazilian packaged foods. However, reductions were inconsistent across food groups, with persistent declarations in natural TFA sources (Group C: meats/eggs/seafood) and refined oil products (Group D: oils/fats/nuts).

This pattern aligns with global studies: Canadian research [14] reported decreased mean TFA content (2013–2017) in NIP, except in bakery products, while Dutch data [15] showed a 48% overall reduction (2006–2016), significant in only 11/27 food categories.

The observed decline suggests industry adoption of alternatives like palm oil, polyunsaturated oils, and interesterified fats [29]. While interesterification is the most studied TFA substitute (among 690 identified options [30]), current evidence remains inconclusive regarding its health effects [31, 32]. These findings highlight the need for improved disclosure of processing methods and replacement ingredients to ensure regulatory compliance and informed consumer choices.

### 4.3 TFA‐Free Declarations and False Negatives

The frequency of TFA‐free claims declined significantly following regulatory changes. Brazil's 2012 reform tightened criteria from ≤ 0.2 g to ≤ 0.1 g TFA/serving, with additional saturated fat restrictions [33]. Subsequent analysis (2010–2013) confirmed this reduced claim usage without consistently affecting i‐TFA content [16]. The 2020 regulation maintained the 0.1 g threshold but switched to a 100 g basis [34, 35], potentially further decreasing claims alongside the PHO ban.

Despite improvements, 24% of 2020 products remained potential false negatives (NIPs showing 0 g TFA but containing i‐TFA terms) ‐ matching findings from 11,434 Brazilian products [36]. This persistent discrepancy highlights ongoing challenges in preventing misleading labels, even after the 2012 and 2019 regulatory changes.

### 4.4 Strengths and Limitations

The present study offers several key strengths: (1) implementation of a standardised, reproducible methodology for identifying i‐TFA terms on food labels, which has been rigorously validated through multiple previous studies by our research group [16, 25, 26, 37, 38]; (2) the methodological framework is explicitly designed for international adaptation, enabling cross‐country comparisons of i‐TFA labelling practices; (3) to our knowledge, this represents the most comprehensive cross‐sectional analysis of TFA labelling in Brazil, both in terms of temporal scope (2010–2020) and product coverage; and (4) the findings provide critical baseline data for monitoring regulatory compliance with Brazil's i‐TFA ban and updated labelling policies, while establishing a robust evidence base for longitudinal assessments.

The study's limitations include its reliance on data from a single supermarket chain; however, this retailer ranks among Brazil's top 15 national chains, enhancing the sample's representativeness. Additionally, for each food label census, types of foods that did not contain at least one product with added fat in their composition were excluded, meaning that the food supply analysed varied across the years. Nevertheless, these exclusion criteria were consistently applied across all food census years to assess the availability of packaged foods that could contain i‐TFA and the potential changes in consumer exposure over time. The sample size varied across the years of analysis, which may have influenced the results. However, the use of logistic and multinomial regression models for data analysis helps mitigate this issue. An ongoing study by the research group will examine matched products using a longitudinal design to isolate the effects of reformulations. Lastly, this paper focused on potential regulatory influences of the food supply, but we acknowledge the presence of time‐varying factors outside of policy changes, such as shifting consumer preferences, which may have also influenced product reformulation during this period. Future research should expand monitoring efforts to assess: (1) post‐2023 regulatory impacts, (2) industry adoption of i‐TFA alternatives and (3) the public health implications of reformulation strategies, thereby informing nutrition policy development.

## 5 Conclusion

While Brazilian packaged foods showed significant declines in i‐TFA declarations (ingredient lists), TFA content (NIPs) and TFA‐free claims between 2010 and 2020, reductions varied across food groups. Despite 2019 mandatory regulations to limit and prohibit i‐TFA, potential i‐TFA terms persisted in 2020, particularly through ambiguous alternative labelling. The i‐TFA limits drove continuing reductions observed from the TFA‐free claim reforms, though full implementation post‐2023 requires continued monitoring to ensure compliance. While manufacturers now use fewer TFA‐free claims, 24% of foods were still classified as potential false negatives in 2020. Persistent false negatives undermine public health goals and mislead consumers relying on label claims. Standardising fat/oil terminology remains critical to prevent consumer misinformation.

This study provides important evidence to support ongoing regulatory discussions in Brazil and offers a basis for evaluating regulatory efficacy. These findings underscore the need for continued monitoring, enforcement of labelling standards and further research to ensure that public health goals regarding i‐TFA elimination are fully executed.

## Author Contributions

Beatriz Vasconcellos de Barros conducted the formal analysis and investigation, with contributions from Mariana Vieira dos Santos Kraemer, Lana Vanderlee and Ana Carolina Fernandes. Mariana Vieira dos Santos Kraemer, Rossana Pacheco da Costa Proença and Ana Carolina Fernandes developed the methodology. Rossana Pacheco da Costa Proença and Ana Carolina Fernandes managed the project. Mariana Vieira dos Santos Kraemer, Lana Vanderlee, Rossana Pacheco da Costa Proença and Ana Carolina Fernandes supervised the research. Elisa Milano, Tailane Scapin, Greyce Luci Bernardo, Maria Cecília Cury Chaddad and Paula Lazzarin Uggioni validated the findings. Beatriz Vasconcellos de Barros wrote the first draft, with all authors reviewing and editing subsequent versions of the manuscript.

## Ethics Statement

The authors have nothing to report.

## Conflicts of Interest

The authors declare no conflicts of interest.

## Supporting information

Supporting File

## Data Availability

The data that support the findings of this study are available from the corresponding author upon reasonable request.
