# Supplementary material for: Evaluating Trans‐Fatty Acids Labelling in Packaged Foods Sold in Brazil Before and After National Policy Changes
Source: J Hum Nutr Diet. 2026 May 10;39:e70253. doi: 10.1111/jhn.70253 (PMC13158319; doi:10.1111/jhn.70253)
Supplement: Supplementary file 1 — Supporting File [file JHN-39-0-s001.docx]

**Title: Analysing trans-fatty acids labelling in packaged foods sold in Brazil in 2010, 2013 and 2020 in the context of food policy changes**

**Supplementary Table I -** Specific terms for sources of industrially produced trans-fatty acids and their frequency of occurrence on the ingredients list of packaged foods sold in Brazilian supermarkets, 2010, 2013 and 2020.

| **Specific terms** | **Years** | | |
| --- | --- | --- | --- |
|  | **2010** | **2013** | **2020** |
|  | **n (%)** | **n (%)** | **n (%)** |
| Hydrogenated vegetable fat | 305 (91,0%) | 228 (85,5%) | 89 (61,4%) |
| Hydrogenated vegetable oil | 8 (2,4%) | 2 (0,7%) | 11 (7,6%) |
| Hydrogenated palm kernel vegetable fat | - | - | 10 (6,9%) |
| Partially hydrogenated vegetable fat | 1 (0,3%) | 8 (3,0%) | 5 (3,4%) |
| Hydrogenated soybean fat | 4 (1,2%) | 2 (0,7%) | 4 (2,8%) |
| Hydrogenated soybean vegetable fat | - | - | 4 (2,8%) |
| Partially hydrogenated soybean vegetable fat | - | - | 4 (2,8%) |
| Hydrogenated fat | 1 (0,3%) | 2 (0,7%) | 3 (2,1%) |
| Hydrogenated vegetable fat from palm | - | - | 3 (2,1%) |
| Hydrogenated coconut and palm kernel vegetable oils | - | - | 3 (2,1%) |
| Hydrogenated palm oil | - | - | 2 (1,4%) |
| Hydrogenated coconut vegetable fat | - | - | 2 (1,4%) |
| Hydrogenated palm fat | - | - | 1 (0,7%) |
| Hydrogenated palm kernel oil | - | - | 1 (0,7%) |
| Hydrogenated canola oil | - | - | 1 (0,7%) |
| Hydrogenated vegetable fat (palm, coconut) in varying proportions | - | - | 1 (0,7%) |
| Liquid and hydrogenated vegetable oils | - | - | 1 (0,7%) |
| Partially hydrogenated soybean and cottonseed oils | - | 5 (1,9%) | - |
| Hydrogenated palm oil | - | 5 (1,9%) | - |
| Vegetable hydrogenated fat | - | 4 (1,5%) | - |
| Partially hydrogenated soybean fat | 2 (0,6%) | 3 (1,1%) | - |
| Partially hydrogenated soybean oil | - | 3 (1,1%) | - |
| Hydrogenated vegetable fat | - | 2 (0,7%) | - |
| Hydrogenated soybean oil | - | 1 (0,4%) | - |
| Hydrogenated vegetable oils | - | 1 (0,4%) | - |
| Partially hydrogenated soybean vegetable oil | - | 1 (0,4%) | - |
| Partially hydrogenated vegetable oil | 6 (1,8%) | - | - |
| Partially hydrogenated/interesterified fat | 2 (0,6%) | - | - |
| Liquid and hydrogenated vegetable oil | 2 (0,6%) | - | - |
| Hydrogenated | 1 (0,3%) | - | - |
| Hydrogenated vegetable margarine | 1 (0,3%) | - | - |
| Hydrogenated corn oil | 1 (0,3%) | - | - |
| Hydrogenated cottonseed, soybean, and palm oils | 1 (0,3%) | - | - |
| **Total per year** | 335 (100,0%) | 268 (100,0%) | 145 (100,0%) |

**Supplementary Table II -** Alternative terms for sources of industrially produced trans-fatty acids and their frequency of occurrence on the ingredients list of packaged foods sold in Brazilian supermarkets in 2010, 2013, and 2020.

| **Alternative terms** | **Years** | | |
| --- | --- | --- | --- |
|  | **2010** | **2013** | **2020** |
|  | **n (%)** | **n (%)** | **n (%)** |
| Vegetable fat | 771 (78,4%) | 728 (63,6%) | 753 (54,1%) |
| Margarine | 177 (18,0%) | 151 (13,2%) | 110 (7,9%) |
| Vegetable palm fat^1^ | - | - | 69 (5,0%) |
| Vegetable cream | 5 (0,5%) | 1 (0,1%) | 67 (4,8%) |
| Dairy-based blend | - | 8 (0,7%) | 32 (2,3%) |
| Milk chocolate | - | 9 (0,8%) | 28 (2,0%) |
| Cream | - | - | 25 (1,8%) |
| White chocolate | - | 10 (0,9%) | 26 (1,9%) |
| Chocolate chips | - | 8 (0,7%) | 24 (1,7%) |
| Milk chocolate chips | - | 4 (0,3%) | 16 (1,1%) |
| Liquid and modified vegetable oils | - | - | 16 (1,1%) |
| Cookie | - | - | 15 (1,1%) |
| Chocolate | - | 5 (0,4%) | 12 (0,9%) |
| Chocolate-flavored chips | - | 4 (0,3%) | 12 (0,9%) |
| Chocolate-flavoured topping | - | - | 12 (0,9%) |
| Dairy-based blend with vegetable fat | 11 (1,1%) | 14 (1,2%) | 11 (0,8%) |
| Seasoning prepared from/flavored/identical to natural flavor...^2^ | - | 42 (3,7%) | 10 (0,7%) |
| Chantilly | - | - | 10 (0,7%) |
| Chocolate sprinkles | - | 1 (0,1%) | 9 (0,6%) |
| Vegetable fats | - | 13 (1,1%) | 8 (0,6%) |
| Chocolate/strawberry/dulce de leche/vanilla flavoured filling | - | 3 (0,3%) | 8 (0,6%) |
| Semisweet chocolate flavour topping | - | 2 (0,2%) | 8 (0,6%) |
| Modified vegetable fat | - | - | 8 (0,6%) |
| Vegetable margarine | 9 (0,9%) | 13 (1,1%) | 7 (0,5%) |
| Semisweet chocolate | - | 6 (0,5%) | 7 (0,5%) |
| Semisweet chocolate chips | - | - | 6 (0,4%) |
| Sprinkles | - | - | 5 (0,4%) |
| Palm vegetable cream | - | - | 5 (0,4%) |
| Coconut vegetable cream | - | - | 5 (0,4%) |
| White chocolate chips | - | - | 5 (0,4%) |
| Chocolate-flavoured biscuit | - | 3 (0,3%) | 5 (0,4%) |
| Powdered palm vegetable fat | - | - | 4 (0,3%) |
| Cottonseed fat and/or palm kernel oil | - | - | 4 (0,3%) |
| Chicken broth | - | 24 (2,1%) | 4 (0,3%) |
| Chocolate flavored sprinkles | - | 3 (0,3%) | 4 (0,3%) |
| Beef broth | - | 10 (0,9%) | 3 (0,2%) |
| Cocoa drops | - | 1 (0,1%) | 3 (0,2%) |
| Mozzarella cheese flavored topping | - | - | 3 (0,2%) |
| Semisweet chocolate flavored drops for topping | - | - | 3 (0,2%) |
| Chocolate blend | - | - | 3 (0,2%) |
| Chocolate /white chocolate/milk chocolate stripes | - | - | 3 (0,2%) |
| Seasoning of…^2^ | - | - | 3 (0,2%) |
| Milk chocolate flavoured topping | - | 4 (0,3%) | 2 (0,1%) |
| Soy vegetable fat | 1 (0,1%) | - | 2 (0,1%) |
| Vegetable fatty acids | - | - | 2 (0,1%) |
| Soybean oil (vegetable fat) | - | - | 2 (0,1%) |
| Chocolate shavings | - | - | 2 (0,1%) |
| Fat | 1 (0,1%) | 2 (0,2%) | 1 (0,1%) |
| Sunflower vegetable fat | 5 (0,5%) | - | 1 (0,1%) |
| Butter oil | - | - | 1 (0,1%) |
| Dairy-based blend with milk-flavored vegetable fat | - | - | 1 (0,1%) |
| Hazelnut cream | - | - | 1 (0,1%) |
| Salted caramel crisp | - | - | 1 (0,1%) |
| Coffee crisp | - | - | 1 (0,1%) |
| Animal and vegetable fat | - | - | 1 (0,1%) |
| Liquid palm fat^1^ | - | - | 1 (0,1%) |
| Vegetable and animal fat | - | - | 1 (0,1%) |
| Vegetable fat and butter | - | - | 1 (0,1%) |
| Chocolate Filling | - | - | 1 (0,1%) |
| *Requeijão**/Creamy *Requeijão** | - | 36 (3.1%) | - |
| Seasoning^2,3^ | - | 15 (1,3%) | - |
| Dark chocolate-flavored stripes^4^ | - | 5 (0,4%) | - |
| Triglyceride mixture^4^ | - | 4 (0,3%) | - |
| Chocolate syrup^4^ | - | 3 (0,3%) | - |
| Organic vegetable fat^4^ | - | 2 (0,2%) | - |
| Marshmallow^4^ | - | 2 (0,2%) | - |
| Hydrogenated vegetable protein | - | 1 (0,1%) | - |
| Hardened olive oil^4^ | - | 1 (0,1%) | - |
| Broiler broth^4^ | - | 1 (0,1%) | - |
| Chocolate-flavored confectionary sprinkles^4^ | - | 1 (0,1%) | - |
| Chocolate-flavored confectionary^4^ | - | 1 (0,1%) | - |
| Vegetable oils and fats^4^ | - | 1 (0,1%) |  |
| Complete seasoning powder^4^ | - | 1 (0,1%) | **-** |
| Seasoning similar to...^2,4^ | - | 1 (0,1%) | - |
| Dairy beverage mix^4^ | 3 (0,3%) | - | - |
| **Total per year** | 983 (100,00) | 1143 (100,00) | 1393 (100,00) |

^1^ Palm and coconut fats were introduced as alternative terms only in 2020, following the identification of at least one product containing partially hydrogenated fat from these sources. They were previously considered trans-fat-free in the 2010 and 2013 guidelines.

^2^ Different flavors (e.g., cheese, ham, sausage, barbecue) were grouped under the same category.

^3^ "Flavor seasoning" and "Cream cheese" were not classified as alternative terms in 2020 because they no longer contained ingredients that may potentially include trans-fats.

^4^ Term not found in the ingredient lists of packaged foods in 2020.

**Requeijão*: Brazilian creamy cheese spread.

**Supplementary Table III** – Logistic regressions of foods with at least one specific or alternative term in the ingredients list while having a TFA-free claim or declared 0 g TFA in the nutrition information panels (false negatives) between 2010, 2013 and 2020 in Brazil.

| **Food groups**  **Years** | **Foods containing >0g TFA declaration on  Nutrition Information Panels (N = 9464)** | | | |  | **Foods containing TFA-free**  **nutrition claims (N = 9900)** | |
| --- | --- | --- | --- | --- | --- | --- | --- |
|  | **All false negatives** | **X^2^**  **p-value** | **False negatives with**  **alternative terms (n=2767)** | **X^2^**  **p-value** |  | **All false negatives** | **X^2^**  **p-value** |
|  | **OR 95% CI** |  | **OR 95% CI** |  |  | **OR 95% CI** |  |
| *All food groups* | | | | |  |  | |
|  |  | **<0,001** |  | **<0,001** |  |  | **<0,001** |
| 2013vs.2010^1^ | 0.72 (0.64; 0.80) |  | 1.16 (0.91; 1.49) |  |  | 0.40 (0.33; 0.48) |  |
| 2020vs.2010^1^ | 0.55 (0.49; 0.61) |  | 2.47 (1.88; 3.25) |  |  | 0.03 (0.02; 0.04) |  |
| 2020vs.2013^2^ | 0.77 (0.69; 0.85) |  | 2.12 (1.61; 2.81) |  |  | 0.07 (0.04; 0.12) |  |
| *Group A - bakery goods, bread, cereals, and related products* | | | | |  |  | |
|  |  | **<0,001** |  | **<0,001** |  |  | **<0,001** |
| 2013vs.2010^1^ | 0.46 (0.37; 0.57) |  | 1.39 (0.87; 2.26) |  |  | 0.34 (0.24; 0.47) |  |
| 2020vs.2010^1^ | 0.46 (0.37; 0.57) |  | 3.85 (2.15; 7.34) |  |  | 0.04 (0.02; 0.08) |  |
| 2020vs.2013^2^ | 1.00 (0.80; 1.25) |  | 2.76 (1.45; 5.52) |  |  | 0.12 (0.05; 0.25) |  |
| *Group B - milk and dairy products* | | | | |  |  | |
|  |  | 0.320 |  | **0.001** |  |  | ***** |
| 2013vs.2010^1^ | 1.21 (0.62; 2.37) |  | * |  |  | * |  |
| 2020vs.2010^1^ | 0.77 (0.42; 1.44) |  | * |  |  | * |  |
| 2020vs.2013^2^ | 0.63 (0.35; 1.17) |  | * |  |  | * |  |
| *Group C - meats, eggs, and seafood products* | | | | |  |  | |
|  |  | **<0,001** |  | **0.032** |  |  | **<0,001** |
| 2013vs.2010^1^ | 0.42 (0.23; 0.75) |  | * |  |  | * |  |
| 2020vs.2010^1^ | 0.13 (0.08; 0.23) |  | * |  |  | * |  |
| 2020vs.2013^2^ | 0.31 (0.19; 0.53) |  | * |  |  | * |  |
| *Group D - oils, fats, and nuts* | | | | |  |  | |
|  |  | **0.008** |  | **<0,001** |  |  | **0.023** |
| 2013vs.2010^1^ | 0.42 (0.21; 0.80) |  | 0.83 (0.26; 2.61) |  |  | * |  |
| 2020vs.2010^1^ | 0.41 (0.23; 0.73) |  | 8.45 (2.63; 31.10) |  |  | * |  |
| 2020vs.2013^2^ | 0.98 (0.57; 1.73) |  | 10.14 (3.09; 38.35) |  |  | * |  |
| *Group E - sugars, sugary foods, and snacks* | | | | |  |  | |
|  |  | **0.049** |  | **<0,001** |  |  | **<0,001** |
| 2013vs.2010^1^ | 0.81 (0.67; 0.97) |  | 1.27 (0.88; 1.84) |  |  | 0.41 (0.31; 0.54) |  |
| 2020vs.2010^1^ | 0.82 (0.69; 0.98) |  | 2.10 (1.44; 3.08) |  |  | 0.03 (0.01; 0.05) |  |
| 2020vs.2013^2^ | 1.02 (0.87; 1.19) |  | 1.65 (1.14; 2.40) |  |  | 0.06 (0.03; 0.12) |  |
| *Group F - gravies, sauces, ready-made seasonings, broths, soups, and ready-to-eat dishes* | | | | |  |  | |
|  |  | **<0,001** |  | 0.560 |  |  | **<0,001** |
| 2013vs.2010^1^ | 0.72 (0.51; 1.00) |  | 0.83 (0.41; 1.69) |  |  | 0.79 (0.40; 1.56) |  |
| 2020vs.2010^1^ | 0.51 (0.37; 0.71) |  | 1.27 (0.60; 2.76) |  |  | 0.07 (0.01; 0.25) |  |
| 2020vs.2013^2^ | 0.71 (0.51; 0.99) |  | 1.53 (0.71; 3.38) |  |  | 0.09 (0.01; 0.32) |  |

^1^ 2010 was used as reference.

^2^ 2013 was used as reference.

* Insufficient number of items in the category for analysis of statistical significance.
